# Circular RNAs: Emblematic Players of Neurogenesis and Neurodegeneration

**DOI:** 10.3390/ijms23084134

**Published:** 2022-04-08

**Authors:** Marianna D’Anca, Francesca R. Buccellato, Chiara Fenoglio, Daniela Galimberti

**Affiliations:** 1 Foundation IRCCS Ca’ Granda, Ospedale Maggiore Policlinico, 20122 Milan, Italy; francesca.buccellato@unimi.it (F.R.B.); or chiara.fenoglio@unimi.it (C.F.); or daniela.galimberti@unimi.it (D.G.); 2 Department of Biomedical, Surgical and Dental Sciences, University of Milan, 20122 Milan, Italy; 3 Department of Pathophysiology and Transplantation, University of Milan, 20122 Milan, Italy

**Keywords:** circular RNAs, Alzheimer’s disease, frontotemporal dementia, Parkinson’s disease, aging, neurogenesis, neurodegeneration

## Abstract

In the fascinating landscape of non-coding RNAs (ncRNAs), circular RNAs (circRNAs) are peeping out as a new promising and appreciated class of molecules with great potential as diagnostic and prognostic biomarkers. They come from circularization of single-stranded RNA molecules covalently closed and generated through alternative mRNA splicing. Dismissed for many years, similar to aberrant splicing by-products, nowadays, their role has been regained. They are able to regulate the expression of linear mRNA transcripts at different levels acting as miRNA sponges, interacting with ribonucleoproteins or exerting a control on gene expression. On the other hand, being extremely conserved across phyla and stable, cell and tissue specific, mostly abundant than the linear RNAs, it is not surprising that they should have critical biological functions. Curiously, circRNAs are particularly expressed in brain and they build up during aging and age-related diseases. These extraordinary peculiarities make circRNAs potentially suitable as promising molecular biomarkers, especially of aging and neurodegenerative diseases. This review aims to explore new evidence on circRNAs, emphasizing their role in aging and pathogenesis of major neurodegenerative disorders, Alzheimer’s disease, frontotemporal dementia, and Parkinson’s diseases with a look toward their potential usefulness in biomarker searching.

## 1. Introduction

Circular RNAs (CircRNAs) have long remained concealed until a new generation of transcriptome techniques have brought them to the fore. Wrongly, they have been overlooked as artifacts of the splicing process, but now they undoubtedly fall into a new subclass of non-coding RNAs (ncRNAs) with significant perspectives [1]. To understand their importance, suffice to remember that only 2% of RNA transcripts encodes for proteins versus more than 60% of transcribed RNAs [2]. This implies crucial biological functions for the noncoding transcriptome. Particularly, circRNAs are evolutionary conserved across eukaryotic tree of life at sequence and expression pattern levels, especially in the brain, demonstrating their ubiquitous functions [3,4]. Additionally, circRNAs show tissue and sometimes cell specific expression. They are enriched in neural tissue so much so that 20% of the cerebral encoded genome produces circRNAs [5]. Furthermore, circRNAs are generated at the expense of regular mRNA transcripts underlying a regulatory mechanism on mRNA amount, acting as “mRNA traps” [6]. Interestingly, a neural specific regulation of circRNA production seems to be because many circRNAs are exclusively expressed in the brain and in specific brain areas or in a higher percentage with respect to other tissues [4]. CircRNAs are particularly present in neurons, where they are localized in the cytoplasm, but many of them accumulate in synapses. Perhaps this is the reason why they seem to be concentrated in the cerebellum, notoriously dense neuronal spines, synapses, and neurons [5]. They probably contribute to neural development as during neuronal differentiation, their number increases, varying their expression temporally and spatially [7]. These findings suggest that circRNAs are crucial for neural function and are required in different neural tissues and developmental processes. Intriguingly, they are differently expressed during synaptogenesis, and in many cases, with no correlation with their linear mRNA isoforms, highlighting singular and specific functions of the circularization events [8]. In view of the above, it is no wonder that circRNAs should play a role in neural aging and neurodegeneration. Indeed, it has been reported in different organisms that during brain aging, circRNAs dramatically increase their expression levels compared to the host genes [7,9,10,11,12]. This is probably due to the resistance to degradation, favoring their concentration in aged tissue as the brain, dense with postmitotic cells [13]. Additionally, malfunction in an alternative splicing process during aging could further implement the circRNA biogenesis in the nervous system [14]. Since numerous works report a progressive increment of circRNAs in the brain during aging, their link to age-related maladies such as Alzheimer’s disease (AD), frontotemporal dementia (FTD), and Parkinson’s disease (PD) appears natural [15]. More importantly, circRNAs are able to cross the blood–brain barrier (BBB) and they are abundant in small extracellular vesicles [16,17]. These two features, combined with their extreme stability to exonuclease action, their abundance in aged neural tissue as well as their selective presence in typical brain cells, make circRNAs a reliable marker of aging diseases to bet on. Thus, the present review aims to elucidate recent knowledge of circRNAs regarding their fundamental role in aging and neurodegeneration with a look toward their potentialities as biomarkers and therapeutic targets.

## 2. Circular RNAs: Biogenesis, Mode of Action, and Functions

### 2.1. Biogenesis

CircRNAs represent a unique class of non-coding RNAs where the ends 5′ and 3′ are covalently joined to form a closed circle structure with a large variety in length and mechanisms of origin [18,19]. More precisely, circRNAs can be derived from exons (ecRNA) or introns (ciRNAs), the predominant class, or both exon-intron (ElciRNAs) transcribed from pre-mRNAs sequences by RNA polymerase II by involving spliceosome machinery [20,21]. Realistically, circRNA production relies on RNA binding proteins (RBPs) and a combination of cis-elements and trans-factors [22]. Actually, most of the circRNAs are generated co-transcriptionally thanks to the escape of lariat RNAs debranching or recircularization of full-length introns with unknown post debranching events for the intronic circRNAs [21]. Instead, for the exonic circRNAs, back-splicing events from constitutive exons come into play, compromising the linear mRNAs, in a kind of competitive mechanism where it would seem that circRNA transcripts are preferred when spliceosome elements or terminator transcription factors are limiting [6,22]. In this case, circRNAs could regulate linear mRNA production, acting as “mRNAs traps”, since many circRNAs are more abundant than corresponding mRNA linear forms [6,23]. On the other hand, there have been several reported cases where there is no correlation among the levels of circRNAs and the linear host mRNA [4,6,10]. One could theorize the coexpression of RBPs that alternatively promote or inhibit circularization as well as the presence of post-transcriptional events that stabilize circRNAs or reduce linear host mRNA half-life [6,23]. Regardless, the circularization and linear alternative splicing regulation mechanisms are still obscure. Exonic circRNAs localize preferentially in cytosol and their nuclear export seems partially based on their length [24]. Likewise, intronic and some exonic cirRNAs preferentially concentrate and function in the nucleus [20]. Additionally, circRNAs can be translocated to other destinations by extracellular vesicles [25]. There is few evidence of the presence of circRNAs in other organelles such as mitochondria that require further and deeper investigations [26].

### 2.2. Mode of Action and Functions

Even if circRNAs’ ubiquitous presence suggests conservative and critical roles in organisms, their functions are still under study. So far, many possible mechanisms of circRNAs action have been proposed. The most accredited is to bind and sequester miRNAs; circRNAs serve as “miRNAs sponges”. In this way, circRNAs hinder miRNA action on target sites and exert a regulatory role on gene expression [27]. CDR1as can be defined as the circRNA “miRNA sponge” par excellence with more than 70 conserved miRNA-7 binding sites; it highly inhibits miR-7 activity [27]. Furthermore, this circRNA is abundant in the brain and its interaction is reported as brain specific [3]. However, it can be seen that not all circRNAs had miRNA binding sites, and other plausible functions have been designated [28]. Indeed, recent works have demonstrated that circRNAs are able to interact not only with miRNAs, but also with proteins exerting multiple effects on them [29]. Interaction patterns of circRNAs with proteins are no doubt more sophisticated and intriguing than those with miRNAs. First, nuclear circRNAs can enhance the transcription of their host genes, binding RNA polymerase II (RNA Pol II) [20]. When they bind regulatory RBPs, they can act as protein sponges, decoys, scaffolds, and recruiters, modulating and influencing many fundamental cellular processes such as cell cycle, apoptosis, senescence, and cell survival, among others [21]. CircRNAs such as protein sponges retain specific proteins in the cytoplasm, preventing their moving into the nucleus. For instance, Circ-Foxo3, sequestering different proteins in the cytosol such as anti-stress transcription factors as inhibitor of DNA binding 1 (ID-1) and E2F Transcription Factor 1 (E2F1) or senescence proteins such as Focal Adhesion Kinase (FAK), Hypoxia-Inducible Factor 1-alpha (HIF-1α) are able to block their anti-senescence and anti-stress functions, enhancing cellular senescence [30]. They can variate the usual protein function, decoying a single specific protein to a specific cellular compartment by stabilizing or sequestering it. In primary neurons of Amyotrophic Lateral Sclerosis (ALS) disease models, intronic circRNAs concentrate in cytoplasm decoying TAR DNA-Binding protein 43 (TDP-43) and avoiding the accumulation of toxic TDP-43 aggregates [31]. CircRNAs can also favor interactions among proteins by working as scaffolds to generate the protein complex. An interesting example is circZNF827, which negatively regulates neuronal differentiation nucleating hnRNP K and L transcription factors to repress NGFR, consequently “tapping a brake” on neurogenesis [32]. CircRNAs can also act as protein recruiters, moving determinate proteins to specific subcellular localizations. The exonic circRNA FECR1 recruits Ten-eleven translocation methylcytosine dioxygenase 1 (TET1), a demethylase, to the Friend leukemia integration 1 transcription factor (FLI1) promoter region, enhancing the transcription of the host gene [33]. This additional function might allow circRNAs translocating RBPs to distant compartments within neurons such as synapses and synaptosome where they regulate synaptic function [4]. CircRNAs were thought untranslatable but considering that most of them originated from protein-coding exons and some contained open reading frame (ORF) and internal ribosome entry site (IRES), it is reasonable to believe that they can encode small peptides [34]. So far, this feature has been demonstrated in vitro and in vivo for a subset of circRNAs, while evidence of endogenous circRNA translation is absent [35,36]. Finally, metabolism of circRNAs is sophistically regulated by RPBs modulating biosynthesis and degradation according to a specific kind of circRNA as well as tissue or cell features and biological conditions. Because different binding elements for RBPs flank circRNAs, this means that the same RPBs can play opposite roles on circRNAs based on which the functional domain is involved. However, the existence of circRNAs can be regulated in different ways. As previously mentioned, circRNA production competes with linear forms via RNA pol II, depending on splicing efficiency. Proliferating cells need linear RNAs more than less proliferating ones, so in the first ones where the splicing should be more efficient, the portion of linear transcripts could overcome the circular ones and vice versa [6]. Furthermore, circRNA expression can be regulated under pathophysiological status since RPB expression varies spatiotemporally. Additionally, several works have shown that RPBs including Adenosine Deaminases Acting on RNA (ADAR), FUS (Fused in Sarcoma), and nuclear factors such as NF90/NF110, RNA-Binding Motif 90 (RBM90) and others can control RNA circularization in different species and experimental designs [37,38,39]. Finally, transcription factors as c-Myb and Twist Family BHLH Transcription Factor 1 (TWIST1) can also exert their function of binding the corresponding promoters of circHIPK3 and Cul2 circRNAs, enhancing their expression [40,41]. The resistance to degradation is a peculiar feature distinguishing circRNAs from linear cognate RNAs. Even if they do not undergo cellular RNA decay machineries, it is still not understood how and how much circRNAs are removed in vivo. It has been speculated that circRNAs degradation is triggered by an endonuclease and then a combined action of exo- and endonuclease occurs. A few studies have demonstrated a global circRNA degradation by RNAse L but also miRNAs and RNAi machinery seem to be involved in circRNAs decay [42,43,44]. In addition, exocytosis or extracellular vesicles can contribute to circRNAs clearance [45]. Furthermore, more studies are needed to better explore the circRNA world.

## 3. Circular RNAs: Role in Neural Development and Aging

The ever-increasing age in the global population has gradually attracted curiosity and interest in studies focused on cellular and molecular mechanisms linked to aging and age-related diseases. Aging is universally defined as relentless functional decline of every organism marked by time. circRNAs’ contribution to this process results from compelling evidence where a progressive accumulation of circRNAs during aging, particularly in the brain, has been demonstrated [3,4,46]. On the other hand, circRNAs are fundamental players already in the early stages of the central nervous system (CNS) development where progenitor cell proliferation and neural differentiation are milestones [4,47]. Neuronal circRNAs are extremely conserved across mammalians such as mouse, pig, and human, and more conserved circRNAs are more expressed than the other ones, suggesting a priority of importance [4,9] for a subset of circRNAs. During neural differentiation, the majority of circRNAs are upregulated [4] and the relative genes of these circRNAs play fundamental roles in neuronal functions such as dendritic mRNA transport and synaptic membrane exocytosis [48,49,50]. As previously mentioned, during the development of porcine brain, different cerebral areas show different circRNAs expression levels, accumulating in the cortex and cerebellum rather than in the brain stem [10]. The same is true for analyzing the multiple phases of neural differentiation where levels and kinds of circRNAs are differently upregulated [4], indicating that there is a timing in the circRNA expression. Furthermore, the same circRNAs can exert its function at different time-points of neural development and in different tissues [10]. Thus, there appears to be a spatial-temporal regulation of circRNAs, suggesting a basic role in neural proliferation and differentiation [4,47]. How circRNAs can contribute in these processes could be explained by investigating the functions of their host genes. Venø et al. [10] found three over-represented host gene related pathways such as Wingless-related integration site (Wnt) signaling, axon guidance, and Tumor Growth factor β (TGFβ) signaling pathways [10]. These pathways highly influence neural differentiation and neuronal migration. In particular, TGFβ is involved in axon and dendrite specification during fetal brain development with a neuroprotective action and by stimulating neural migration [51,52]. Wnt signaling has a crucial role in the differentiation of progenitor cells and generation of neural circuits while the axon guidance pathway regulates synaptogenesis through axons and dendrites [53,54]. Concerning the latter aspect, the relevance of cirRNAs in synaptogenesis is also indicated by their cellular localization, mainly in synaptoneurosomes, and it is not related to their linear mRNA transcripts, present instead in cytoplasm [5]. Studies on animal models have showed that the brain harbors the major amount of circRNAs regulated during development where the number increased with age, demonstrating a link between circRNAs and aging. Indeed, there was a correlation between circRNAs accumulated during life span and their mRNA targets, revealing that these circRNAs could modulate aging processes mainly through sponging miRNAs [7,55]. This is also confirmed from other evidence, where the circRNA age-accumulation was confined to neural tissue while other organs showed unchanged global circRNA levels [11,56]. The reason why circRNAs accumulate during aging and primarily in the brain is under investigation as well as the underlying mechanisms, although it is thought that this phenomenon is not age dependent specific, but a general age accumulation trend. Referring to the aging process, cellular senescence undoubtedly represents a critical component. Analyzing the circRNAome in peripheral blood of aged people, Haque et al. [57] found a differential expression for circFOXO3 and circEP300 (Table 1) [57].

Interestingly, the same circRNAs also appeared dysregulated in several human senescent cells and were associated with measures of parental longevity (Table 2) [57].

This means that circRNAs can have a role in cellular senescence as well as in cellular survival. In addition, the analysis of pathways most represented in age associated circRNAs showed important differences with an enrichment of genes involved in phagocytosis, circadian regulation, cancer pathways, and Golgi-associated vesicles in elderly donors compared to young ones, meaning that circRNAs could define the aging phenotypes [57].

A previous work also explained that circFOXO3 interacting with Cyclin-Dependent Kinase Inhibitor 1 (p21) and Cyclin Dependent Kinase 2 (CDK2) proteins arrested cell cycle progression and cell proliferation, as it occurs in senescent cells (Table 2) [74]. Forkhead Box O3 (FOXO3) has been raised in several works for its neurotoxic role Aβ-induced promoting neuronal death and mitochondrial dysfunction in AD mice [91,92]. At the opposite, other works have instead proposed a neuroprotective role for FOXO3, for example, in combination with TREM2, reducing the inflammatory response and cognitive impairment [93]. Regardless, this transcription factor seems strongly related to aging because some evidence indicates it to be associated with life span and healthy aging counting among candidate “longevity genes” [94,95]. Curiously, RNA circularization may be associated with a change in function. Muniz et al. [96] observed that the lncRNA ANRIL, which usually repressed senescence by acting on INK4 family genes, could become a senescence activator, switching in circular isoforms under oncogene-induced senescence conditions [96]. The link between circRNAs and senescence led to the emergence of a new subclass of circRNAs named as SAC-RNAs, in which circRNAs are differentially expressed in senescent rather than proliferating cells. The first identified was circPVT1, originating from the lncRNA PVT1 (Table 2). When this circRNA was silenced in proliferating fibroblasts, it triggered senescence, probably by interfering with let-7 activity and consequently let-7-targeted mRNAs [75]. Furthermore, in a very recent work performed on tendon stem/progenitor cells, the same circRNA repressed senescence by sponging mir-199a-5p [76]. This microRNA target Sirtuin1 (SIRT1), a crucial anti-aging factor extremely conserved across phyla, has been shown in compelling evidence to be highly involved in neurodegenerative disorders such as AD, PD, and tauopathies [97]. SIRT1 levels were lower in AD brains and correlated with tau aggregate accumulation [98]. Moreover, in mouse models of AD and FTDP-17, SIRT1 deficiency increased tau acetylation, exacerbating NFT formation [99]. Another interesting SAC-RNA is circCCNB1 generated from two exons of the circularization of cyclin B1 gene (CCNB1) (Table 2). Yu and coworkers [77] discovered that circCCNB1 sponged miR-449a and prevented its binding on the cyclin E2 gene (CCNE2), delaying cellular senescence in vitro [77]. It is worth to count among the circRNAs involved in the senescence process, the circRNA generated by the DNMT1 gene, responsible for DNA methylation stability and is highly expressed in the adult brain (Table 2). In vitro experiments demonstrated that ectopic expression of circDNMT1 increased autophagy and inhibited senescence [78]. Epigenetic changes such as aberrant DNA methylation have been raised as additional molecular risk factors in different neurodegenerative diseases. Several articles have reported a global DNA hypermethylation in subjects with late onset AD (LOAD), the major frequent form of dementia in aged people, associated with an increase in DNA MethylTransferase1 (DNMT1) gene expression and protein amount [100,101,102]. These studies are in contrast with others where a common decrease in DNA methylation characterized AD and PD [103]. Indeed, in PD and related disorders, DNMT1 deregulation appears to be closely associated with pathogenesis in which α-synuclein blocks DNMT1 into the nucleus, leading to a general DNA hypomethylation [104]. In summary, due to the relationship among cellular senescence and aging/age-related diseases and consequently the link between circRNAs and senescence, targeting circRNAs might be a valid strategy for therapeutic intervention on aging and neurodegenerative diseases. Moreover, since the age-related increase in circRNAs seems to be a preserved process, it is conceivable that they could have utility as biomarkers in senescence and aging.

## 4. Circular RNAs: Potential Roles in the Pathogenesis of Neurodegenerative Diseases

As aforementioned, the significant presence of circRNAs in the brain compared to other organs leads to the belief that circRNAs should exert prince roles in the CNS neurobiology as well as neuropathology of neurological disorders including AD, FTD, and PD.

### 4.1. Alzheimer’s Disease (AD)

In search of hypothetical roles of circRNAs in neurodegenerative diseases, most studies have focused on AD. Alzheimer’s disease is the first cause of dementia and affects over 50 million people worldwide, representing a rising challenge for public health care worldwide [105,106]. The disease is irreversible and clinically leads to memory loss, intellectual disabilities, and changes in personality and behavior [107]. The neurodegeneration found in AD is principally due to toxic aggregation of extracellular amyloid plaques and intracellular neurofibrillary tangles (NFTs) of hyperphosphorylated tau protein [108]. Indeed Aβ-amyloid (Aβ), total and phosphorylated tau proteins in the cerebrospinal fluid (CSF) are the main fluid-based biomarkers recognized and dosed in clinical practice [109]. The pioneering study that demonstrated a direct involvement of CDR1as with the pathogenesis of AD is dated back to 2013 (Table 1) [58]. CDR1as/CiRS-7 highly expressed in the human brain acts as a sponge of mir-7, but resulted in being downregulated in AD sporadic patients. The presence of upregulated mir-7 due to the absence of ciRS-7 “sponging” effects can downregulate AD-relevant targets such as the ubiquitin protein ligase A (UBE2A) (Table 1) [59]. This phagocytic protein, essential for amyloid peptide clearance, is depleted in the AD brain and its deficiency contributes to beta-amyloid deposition and the formation of senile plaques [110,111]. The existence of a misregulated ciRS-7/miRNA-7/UBE2A circuit in sporadic AD subjects was next proven by Zhao and colleagues [60], where the reduced levels of UBE2A protein and the increased expression of mir-7 were caused precisely by the ciRS-7 deficit [60]. Interestingly, Shi and coworkers [79] proposed a neuroprotective role for the same circRNA by promoting Amiloid Precursor Protein (APP) and Beta Secretase 1 (BACE1) protein degradation via the proteasome and lysosome pathways (Table 2) [79]. In particular, in human SH-SY cells, the overexpression of ciRS-7 reduced the Aβ production because it inhibited translation of NF-kB and induced its cytoplasmic localization. This led to the increased expression of ubiquitin carboxyl-terminal hydrolase L1 (UCHL1), which promotes APP and BACE1 degradation. All of this evidence testifies to a potential regulatory role for ciRS-7 in the etiology of AD [79]. Similarly, for circHDAC9, a neuroprotective role is reported (Table 2). The upregulation of this circRNA alleviated Aβ(42)-induced neuronal damage in human neuronal (HN) cells by sponging mir-142-5p [80]. It is worth noting that this effect was due to berberine treatment, which enhanced circHDAC9 expression and preserved HN cells from Aβ-42 neuronal damage [80]. This result finds confirmation in the literature where a positive role of berberine in neurodegenerative diseases has been reported, further testifying to the neuroprotective properties [112,113]. In the work of Lu et al. [61], an opposite role was proposed for the same circRNA (Table 1) [61]. circHDAC9 was present at low levels in the serum of MCI and AD patients as well as in vitro and in vivo models of AD. In detail, the authors found that the circuit circHDAC9/miR-138/Sirt1 rescued the inhibition of ADAM10/mir-138-induced increasing levels of Aβ protein and contributed to AD development [61].

The relevance of circRNAs in AD also lies in the cerebral genes from which they were spliced. The circRNA in RTN4 gene, which inhibits neuronal sprouting and modulates AD by reducing the Aβ deposit through interaction with BACE1 and the circRNA in the Homer Scaffold Protein 1 (HOMER1), key gene of postsynaptic regulation, was validated in different transcriptomic studies (Table 1). Both circRNAs, circRTN4 and circHOMER1, in a circular-transcriptome-wide analysis of Dube et al. [62] performed on AD brains, were found to be significantly associated with AD diagnosis, clinical neurological staging, and dementia severity [62]. The same results were also obtained in an independent cohort of familial and sporadic AD subjects with a prominent altered expression in the familial group compared with the sporadic one [63]. In particular, circHOMER1, generated from the HOMER1 gene, is very interesting considering that HOMER1 protein contributes to the postsynaptic density (PSD) by linking among neural channels and receptors with which the Aβ protein in the AD brain can aberrantly combine [114,115]. In the works of Dube [62] and Cervera-Carles [63], circHOMER1 was downregulated in different brain regions while Urdánoz-Casado et al. [64] showed a gender-dependent dysregulation for it with a reduced expression in the entorhinal cortex of only women with AD, speculating a female-specific role for circHOMER1 in AD [64]. Actually, circHOMER1 might be directly related to AD regulating Presenilin 1 (PSEN1) and Presenilin 2 (PSEN2) expression by binding its predicted sites for mir-651 [116]. Not only circ-HOMER1, but also circCORO1C, could have good potential to be counted as novel markers of AD risk and diagnosis. Indeed circCORO1C, likewise circHOMER1, was highly associated with neuropathological AD status vs. controls and other AD severity traits investigated in Dube and colleagues’ research (Table 1) [62]. Furthermore, circCORO1C co-expressed with APP and Sinuclein Alpha (SNCA) AD-related genes. This co-expression could be mediated through mir-105 and its predicted targets, the APP and SNCA genes [116]. Another circRNA generated from the AD-associated gene Dedicator Of Cytokinesis 1 (DOCK1) is reported in several articles (Table 1) [62,65]. DOCK1 participates in axonal outgrowth, spine morphogenesis, and neuroinflammation and was identified as the best gene AD-related in astrocyte, language, and cognitive specific modules of polygenic risk scores combined with brain expression profiles [62,117]. circDOCK1 was detected as more abundant in AD brains compared to HC in the analysis of Dube (2019) and Cochran (2021) [62,65]. Furthermore, Cochran’s analysis also demonstrated differential circRNA expression in blood isolating circKIF1B and circDLG1, whose genes are implicated in vesicular trafficking, to be good biomarker candidates because they appeared both brain and plasma samples (Table 1) [65].

Undoubtedly, the most intriguingly characteristic of some circRNAs is that, as already pointed out, when they contain exonic regions, they could be translated into peptides [35,36]. In this respect, the presence of a circRNA, circ_0007556, encoding the new Aβ-175 polypeptide variant, named as circAβ-a, seems to represent the most direct connection between circRNAs and AD pathogenesis (Table 1) [66]. This circRNA is generated from the circularization of some exons of the APP transcript. Mo et al. [66] detected circAβ-a in both the brains of sporadic AD patients and controls and demonstrated, using in vitro models, that this circRNA was efficiently translated into an Ab-related protein (Ab-175) and was further cleaved into Aβ-peptides, a hallmark of AD [66]. This research shed light on a novel path in the aβ biogenesis, particularly in AD sporadic cases where this process is still elusive, revealing a new potential therapeutic target for AD treatment.

Neuroinflammation, mediated by glia activation, certainly plays a pivotal role in AD progression, contributing to neuronal injury [118]. Therefore, a variety of circRNAs have been supposed to be involved in neuronal inflammation and damage of AD patients or AD-like models. One of the few studies performed on CSF samples of AD samples versus controls correlated the circRNA expression profile with disease and risk progression (Table 1) [67]. Li et al. [67] discovered that circLPAR1, circAXL, and circGPHN could predict higher AD risk, whereas circPCCA, circHAUS4, circKIF18B, and circTTC39C could predict lower AD risk. These circRNAs can modulate the transcription of their originating genes in a positive or negative manner such as AXL receptor tyrosine kinase (AXL) or Tetratricopeptide Repeat Domain 39C (TTC39C), and increase the AD susceptibility by dysregulating neuroinflammation and neuronal cell apoptosis [67]. In addition, circ-PCCA decreased in the CSF of AD patients, which could be directly involved in AD pathogenesis since its overexpression reduced the AD severity by sponging mir-138-5p and inhibiting Tau phosphorylation, a histopathological hallmark of AD [67]. The analysis of Li et al. also highlighted circ-LPAR1, which interestingly, Wu et al. found was highly expressed in AD patients. In this elegant study, they explored the underlying regulatory axis of circLPAR1, explaining how this circRNA can promote Aβ-induced neuronal injury. In detail, circLPAR1 sponged on mir-212-3p led to upregulation of its target ZNF217 and sped up apoptosis, inflammation, and oxidative stress triggered by Aβ25-35 in vitro. Indeed, expression levels of mir-212-3p in AD patients decreased, whereas Zinc Finger Protein 217 (ZNF217) expression increased [68]. Moreover, the involvement of this zinc finger protein in AD was shown in several recent articles, where the regulation of the lncRNA/miRNA/ZNF217 axis modulated the Aβ-induced cell injury [119,120]. In the research of Yang et al. [81], the circ_0000950 appeared directly involved in neuroinflammation since by sponging mir-103, it led to the expression increase of a proinflammatory gene, prostaglandin-endoperoxide synthase 2 (PTGS2) in two different in vitro AD models (Table 2). This circRNA in several experiments enhanced neuronal apoptosis and inflammation while it reduced neurite outgrowth in AD [81].

Autophagy dysfunction represents an early neuropathological feature of AD that can affect the metabolism of Aβ and accumulation of protein Tau [121]. CircRNAs with a regulatory role in the autophagy process have been described in AD-like models (Table 2). In SAMP8 mice, the mmu_circRNA_017963 was highly associated in different autophagosome and vesicular transport pathways [82]. In the same mouse model, circNF1-419 increased autophagy, reducing the expression of AD markers such as Tau, p-Tau, Aβ1-42, and APOE, and ameliorated senile dementia by binding Dinamin-1 and Adaptor protein 2 B1 (AP2B1), influencing multiple signaling pathways, especially at the synapse [83]. The authors proposed the potential therapeutic and diagnostic value of using circNF1-419 in dementia, even if the mechanisms involved and the precise regulatory role of circRNAs in autophagy processes are still scarce and more investigations are needed.

The high levels of reactive oxygen species in the brain exposes it to oxidative damage that affects the amyloidogenic pathway, exacerbating AD progression [122]. The involvement of circular RNA in oxidative stress AD-associated has only recently emerged. The study of Huang et al. [84], although with some limitations, is unique in that it provides a therapeutic agent to target potential circRNA candidates against AD (Table 2). The authors found that mmu_circRNA_013636 and mmu_circRNA_012180 were significantly upregulated and downregulated, respectively, in SAMP8 untreated mice. This condition was exactly reverted under Panax Notogingseng Saponins (PNS) treatment [84]. Different software used to investigate the pathways in which these two circRNAs were involved, and disclosed several AD-related biological mechanisms and pathways, suggesting that mmu_circRNA_013636 and mmu_circRNA_012180 could be implicated in AD neuropathology. However, their participation in AD pathology remains obscure. Previous studies of the same group showed that PNS administration in SAMP8 mice caused a general improvement in AD progression, reducing Aβ deposition, increasing the expression of antioxidant proteins and ameliorating learning and memory status. Therefore, PNS seems to have protective properties against neural oxidative damage and could represent an emerging candidate for AD therapy [123].

The rising interest in RNA biomarkers of neurodegenerative diseases also led us to explore the circRNA world in the effort to find diagnostic and/or prognostic biological markers of pathology. A circRNA supposed to be a potential biomarker of AD, especially because its best matched target is IGF2R, is circ_0131235 (Table 1). This circRNA was increased in the middle temporal (MT) cortex of a well-characterized cohort of AD people with no significant correlation with cognitive ability [69]. IGF2R, the insulin like growth factor 2 receptor, is directly involved in cerebral insulin signaling, and together with INS and INSRR genes, is counted among the LOAD risk genes [101]. In addition, IGF2/IGF2R reduced the amyloid burden and ameliorated memory functions in AD model mice [124]. In particular, the IGF2R gene and its soluble factor are closely associated with diabetes mellitus type 2, a known high risk factor for AD onset [125,126]. A very recent potential candidate AD biomarker is circTulp4. This circRNA, enriched in the brain, is downregulated in APP/PS1 mice and interacts with TUB Like Protein 4 (Tulp4), regulating neuronal growth and differentiation (Table 2). The authors tried to describe a working model in which circTulp4, residing in the nucleus, would form a U1 snRNP and RNA polymerase II complex and influence Tulp4 expression. Indeed, in cerebral tissues, circTulp4 and Tulp4 have comparable expression and when downregulated, affect neurite growth, differentiation, and cell viability. Authors have speculated that this regulatory implication for circTulp4 underlines a hypothetical relationship between this circRNA and AD pathogenesis [85].

### 4.2. Frontotemporal Dementia (FTD)

Frontotemporal dementia (FTD) is the second cause of younger dementia, affecting up to 20% of subjects before 65 years. It comprises a cluster of different neurological syndromes and presentations, but most patients show alterations of behavior and personality with cognitive and executive impairment, except for the language subtypes featured in a prominent language impairment [127]. FTD at the neuropathological level is represented by frontotemporal lobar degeneration (FTLD) and classified on protein-based inclusions into FTLD-Tau, FTLD-TDP 43, and FTLD-FUS [127]. FTD presents high inheritance with more than half of the cases showing a familial history of disease mainly due to mutations in microtubule-associated protein tau (MAPT) and progranulin (GRN) genes, and hexanucleotide (GGGGCC) repeat expansion (HRE) in the first intron of the Chromosome 9 Open Reading Frame (C9ORF)72 [128]. Although studies on this topic are still scarce, it is worth bearing in mind that the MAPT locus generates circRNAs and more importantly, as above-mentioned, there are several RBP proteins including FUS that are involved in circRNA circularization [37,129]. The RBP FUS is known to act in splicing regulation and mutations of this protein could be causally related to FTD pathogenesis [130,131]. The work of Errichelli et al. [37] revealed that FUS regulates and controls circRNA expression by activating or repressing splicing in mouse models [37]. This pioneering study demonstrates that FUS is directly implicated in the splicing regulation of this new class of RNAs and paves the way to elucidate the circRNAs’ role in the pathogenesis of neurodegenerative diseases in which FUS is mainly involved such as FTD and ALS.

The concept that circRNAs could also play a prominent role in FTD was raised for the first time by Cervera-Carles and colleagues’ study [63], where two circRNAs, circHOMER1 and circKCNN2, were significantly decreased in a group of FTLD-Tau and FTLD-TDP43 brain samples (Table 1) [63]. These authors, already mentioned in the AD section, also found a downregulated expression for the same circRNAs in AD patients compared with the controls [63]. These data brought to light that this peculiar kind of RNA could participate in the development of other forms of dementia.

As stated above, the expansion of short nucleotide repeats (GGGGCC) in intron 1 of the C9ORF72 gene is the most common genetic cause of FTD. The pathogenicity of HRE is due to the formation of nuclear RNA granules, generated from repeat-containing spliced intron and unspliced pre-mRNA, with probable disruption of RNA processing and the unconventional translation (RAN) of the GGGGCC repeats into aggregating and insoluble toxic dipeptide repeat (DPR) proteins [132,133,134]. Wang and coworkers [135] demonstrated that spliced introns were stabilized in circular form and exported from the nucleus to cytoplasm where they produced DPR proteins following RAN translation [135]. Their pivotal discovery certainly opens up a new world for the implications of circRNAs also in FTD. Indeed, the authors explained that the G-rich repeats are responsible for the stabilization in the circular form, precisely thanks to the aforementioned lariat debranching escape phenomenon, which characterizes the biogenesis of intronic circRNAs [21].

### 4.3. Parkinson’s Disease (PD)

Parkinson’s disease (PD) is the second most common neurodegenerative disorder of elderly people after AD [136]. It is clinically recognized by motor deficit due to the death of more than 50% of dopaminergic neurons in the substantia nigra-striatum (ST), but cognitive decline can also occur during the course of the disease [137]. The deposition of misfolded α-synuclein proteins that aggregate in large cytoplasm inclusions, named Lewy bodies (LB), represent the neuropathological hallmark of PD [138]. Most PD cases are sporadic, but 5–10% of PD are familial, related to the mutation in autosomal-dominant genes (SNCA, LRKK2, and VPS35) and autosomal recessive genes (PINK1, DJ-1, and Parkin) [139]. However, although several studies support that aberrant RNA metabolism could have a role in the pathogenesis of PD, the circRNA implication in this regard has rarely been investigated in PD [86,140].

Interestingly, the work of Hanan et al. [70] demonstrated that the healthy ST system presented an age-related increasing of circRNA expression that is lost in PD individuals (Table 1) [70]. Curiously, these results were not repeated in other brain regions such as the medial temporalis gyrus (MTG) and the amygdala (AMG), with no difference between healthy or disease samples. The circRNA profiling differentially expressed (DE-circRNAs) among PD and healthy controls detected that circSLC8A1 was significantly upregulated in PD brains. An excess of oxidative stress in the ST system could be one of the possible reasons for dopaminergic neuron damage [141]. Indeed, the circSLC8A1 increase was shown as dose-dependent when SH-SY neuronal cells were exposed to the Paraquat (PQ) oxidative reagent while the Solute Carrier Family 8 Member A1 (SLC8A1) protein levels decreased. This Ca++/Na++ transporter has been identified as a factor that could promote neurodegeneration and neuroinflammation in PD mouse models [142]. Similarly, validated targets of miR-128, which has seven potential binding sites within the circSLC8A1 sequence, were upregulated in both the ST system of PD brains and PQ treated cultured neural cells. Among the mir-128 targets, there is SIRT1, which, as above-mentioned, is not only involved in aging, but it shows anti-inflammatory and neuroprotective effects in neuroinflammation-related diseases such as PD [143]. Furthermore, not only mir-128, but also mir-132, could bind circSLC8A1. This miRNA was altered in PD and regulated pivotal mechanisms associated with PD including dopaminergic neuronal maturation [144].

The implication of circRNAs in oxidative stress also emerged from another work (Table 1) [71]. Even if performed on the peripheral blood of only four PD patients vs. the controls, Kong and colleagues [71] found a general deregulation for circRNAs with 129 circRNAs upregulated and 282 circRNAs downregulated [71]. The top 10 circRNAs dysregulated included circHBB, circSIN3A, and circFBXW7, which are encoded by genes implied in the oxidative stress response. The authors concluded that the profiling of circRNAs in peripheral blood not only provides new insights in PD pathogenesis, but also gives a conspectus of potential disease biomarkers.

From this point of view, Zhong and coworkers [72] analyzed the potential ability of cell-free circRNAs to predict PD at early diagnosis PD and progression (Table 1) [72]. The microarray-based analysis on plasmatic circRNAs of three PD patients vs. the controls found that the circ_0004381 and the circ_0017204 (named as circARID1B and circTCONS-l2-00002816) could predict PD at early diagnosis with relatively high sensitivity and specificity according to risk score formulas and ROC curve analysis. Instead, circ_0085869, circ_0004381, circ_0017204, and circ_0090668 (named as circFAM83H, circARID1B, circTCONS-l2-00002816, and circHUWE1) may be able to discriminate late stage PD from early stage PD. In summary, the cell-free circRNA panel could be used in the research of biomarkers for the early diagnosis or dynamic monitoring of PD progression [72]. Ravanidis et al. [73] also investigated the utility of circRNAs as biomarkers on a wider cohort of PD patients (Table 1) [73]. The authors detected six out of eighty-seven brain enriched circRNAs significantly downregulated in PBMCs of PD patients vs. healthy controls: MAPK9_circ_0001566, HOMER1_circ_0006916, SLAIN1_circ_0000497, DOP1B_circ_0001187, RESP1_circ_0004368, and PSEN1_circ_0003848. Interestingly, most of these circRNAs were hosted by genes with essential functions in brain homeostasis or directly correlated with neurodegenerative diseases. For example, circ_0003848, hosted by PSEN1, is a known causative gene for early onset AD and was recently identified as a possible cause of early onset PD or HOMER1, which has already been mentioned for its implication in AD pathogenesis, seems to be associated with psychotic symptoms of PD [145,146,147]. There are also genes indispensable in neuronal sprouting and brain development such as Mitogen-Activated Protein Kinase 9 (MAPK9) or SLAIN Motif Family Member 1 (SLAIN1), important for axon elongation by promoting persistent microtubule growth [148,149]. Intriguingly, the authors also investigated the potential RBPs and miRNAs with which these DE-circRNAs could act using in silico approaches. Among the RBPs detected, they found fragile X mental retardation 1 (FMR1), Ataxin 2 (ATXN2), FUS, and TDP43 already involved in familial neurodegeneration, while the circRNA-miRNA network analysis revealed miR-659-3p, which targets progranulin, as potentially sponged by at least three DE-circRNAs. As aforementioned, mutations in progranulin genes are associated with familial cases of FTD [150]. Finally, the authors evaluated the diagnostic utility of DE-circRNAs with ROC curve analysis. Four circRNAs (SLAIN1_circ_0000497, SLAIN2_circ_0126525, ANKRD12_circ_0000826, and PSEN1_circ_0003848) had 75.3% sensitivity and 78% specificity in distinguishing PD from healthy donors.

A more extensive study of circRNA expression profiling was performed on different brain regions in a PD mouse model (Table 2). This analysis revealed different circRNA expression profiling and different pathways involved based on the different brain regions analyzed [87]. In detail, the authors found 24, 66, 71, and 121 differentially expressed circRNAs (DE-circRNAs) in the cerebral cortex (CC), hippocampus (HP), striatum (ST), and cerebellum (CB), respectively. These DE-circRNAs were included in several biological functions and molecular mechanisms related to neuron differentiation, PD, axon guidance as well as the cGMP-PKG, and PI3K-Akt signaling pathways. Moreover, the predicted target gene analysis of DE-circRNAs showed that mmu_circRNA_0003292, mmu_circRNA_0001320, mmu_circRNA_0005976, and mmu_circRNA_0005388 targeted PD-associated genes, in particular, mmu_circRNA_0003292 regulated the Nuclear Receptor Subfamily 4 Group A Member 2 (NR4A2) expression sponging miR-132. Previously evidence has shown that mir-132 regulated embryonic stem cell differentiation into dopamine neurons precisely by inhibiting NR4A2 expression [151]. NR4A2 is an important transcription factor, strongly expressed in dopaminergic neurons and is essential for their development and function maintenance. This means that NR4A2 dysregulation may be implicated in the pathogenesis of different neurological diseases such as PD [152].

circRNAs could have also a neuroprotective role in the PD etiology. When circDLGAP4 was inhibited in in vitro PD models, there was viability and autophagy reduction, apoptosis, and mitochondrial damage induction (Table 2) [88]. All of these conditions were reverted when circDLGAP4 was overexpressed. Indeed, these positive effects were lost in PD animal models where its expression was decreased. Investigating molecular interactions of circDLGAP4, the authors confirmed that circDLGAP4 is involved in PD by sponging miR-134-5p. This miRNA was identified as a neurotoxic factor in several neurodegenerative disease such as AD or cerebral ischemia [153,154] by directly targeting cAMP-response element binding protein (CREB) signaling. CREB protein is a neuroprotective transcription factor that promotes the transcription of numerous genes including the Brain Derived Neurotrophic Factor (BDNF), the apoptotic suppressor B-cell lymphoma 2 (Bcl-2), and the regulator of anti-oxidant enzyme such as Peroxisome proliferator-activated receptor gamma coactivator 1-alpha (PGC-1α). These genes are known to participate in neurodegenerative diseases such as AD, ischemic stroke, and PD [155,156,157].

Deregulated apoptosis and autophagy disturbances are phenomena associated with a plethora of neurodegenerative disorders including PD [158]. circSAMD4A was found to participate in apoptosis and autophagy processes in PD animal models (Table 2). The authors identified miR-29c-3p as a target of circSAMD4A, whose expression was upregulated in vitro and in vivo PD models while miR-29c-3p was downregulated [89]. In parallel, circSAMD4A knockdown inhibited apoptosis and autophagy by acting on the AMPK/mTOR cascade, which has been reported to be associated in these two fundamental processes [159].

The study of Sang et al. is noteworthy, since for the first time, we report on the function in PD of circSNCA, hosted by the SNCA gene that encodes the α-synuclein protein (Table 2) [90]. The researchers found that SNCA and circSNCA expression was drastically reduced after treatment with pramipexole (PPX), a dopamine D2/D3 receptor agonist commonly used in PD therapy. Intriguingly, the authors hypothesized that circSNCA was an endogenous competitor for mir-7 binding with the SNCA transcript in PD models since levels of miR-7 were inversely related to circSNCA expression. Additionally, the circSNCA knockdown, leading to SNCA gene downregulation, was associated with apoptosis reduction and autophagy increase [90]. Therefore, circSNCA inhibition could delay worsening of disease and could serve as a potential therapeutic target of PD.

## 5. Conclusions

Given the growing interest in RNA biomarkers for neurodegenerative diseases, circRNAs could represent reliable and affordable candidates. Evidence in support of this is manifold. First, unlike linear RNAs, their circular structure endows them with high RNase resistance and with peculiar structural conformations, distinguishing them from linear RNAs. More importantly, they are present in blood circulation, raising their possibility of being useful not only as disease biomarkers, but also as targets of molecular therapies. In addition, they accumulate, especially in the brain, in an age-dependent manner, and this aspect makes them even more appealing for neurodegenerative biomarker research. Even though the search for specific circRNA biomarkers in neurodegenerative diseases is still in his infancy, the observation that differentially expressed circRNAs in the brain overlaps with the ones in the plasma of patients affected with neurodegenerative diseases has led to the promising perspective of their potential use as peripheral biomarkers. Certainly, circRNAs have attracted general interest for their inheritable stability, their brain abundance, their ability to cross the blood–brain barrier, their highly tissue specific expression, and are not related to their linear cognate RNAs. Their differential expression in disease-associated genes suggests that they also represent crucial determinants of pathophysiological processes implicated in neurological disorders. Not least, the crucial discovery that some circRNAs can be translated into peptides paves the way for new perspectives of the genomic “dark matter” that should not be strictly classified as coding or non-coding. On the other hand, not all that glitters is gold, since circular RNAs can be the result of technical artifacts, and methods to validate the circularity of RNAs are required. Nevertheless, as more and more evidence has brought out the increased power and potentiality of circRNAs, a deeper understanding of their molecular mechanisms in physiological as well as pathological conditions remains warranted.

## Figures and Tables

**Table 1:** circRNAs in aging, Alzheimer’s disease, Frontotemporal Dementia, and Parkinson’s disease.

CircRNA Name	miRNA Sponge or Mechanism of Action	Target	Possible Pathogenic Role	Tissue or Body Fluid	Potential Disease Biomarker	Host Gene	References
Aging							
circFOXO3, circEP300				Blood		Forkhead box 3, E1A binding protein p300	[57]
Alzheimer’s disease							
ciRS-7	mir7	UBEA2	β-amyloid deposition degradation	Brain		Cerebellar degeneration-related protein 1 antisense RNA	[58,59,60]
circHDAC9	miR138	Sirt1	Increase levels of Aβ protein	Serum		Histone deacetylase-9	[61]
circHomer1	mir-651	PSEN1 and PSEN2	Postsynaptic regulation	Brain entorhinal cortex	Yes	Homer scaffold protein 1	[62,63,64]
circRTN4			Inhibits neuronal sprouting, reduce Aβ deposition through BACE1		Yes	Reticulon-4	[62,63]
circCORO1C	mir-105	APP, SNCA		Brain	Yes	Coronin 1	[62]
circDOCK1			Axonal outgrowth, spine morphogenesis, neuroinflammation	Brain, plasma	Yes	Dedicator of cytokinesis 1	[62,65]
circKIF1B circDLG1			Axonal transport, vesicular traffic	Brain, plasma	Yes	Kinesin family member 1B, Discs large MAGUK scaffold protein 1	[65]
circAβ-a				Brain	Yes	Amyloid-β-a	[66]
circLPAR1	Modulate transcription/mir-212-3p	PPAR1 ZNF217	Neuroinflammation, neuronal cell apoptosis/oxidative stress	CSF, blood	Yes	Lysophosphatidic acid receptor 1	[67,68]
circAXL, circGPHN	Modulate transcription	AXL, GPHN	Neuroinflammation, neuronal cell apoptosis	CSF	Yes	AXL receptor tyrosine kinase, Gephyrin	[67]
circPCCA	mir-138-5p		Inhibits Tau phosforilation	CSF	Yes	Propionyl-CoA carboxylase subunit alpha	[67]
circ_0131235		IGF2-receptor		Brain	Yes		[69]
Frontotemporal dementia							
circHomer1				Brain		Homer scaffold protein 1	[63]
circKCNN2				Brain		Potassium calcium-activated channel subfamily N member 2	[63]
Parkinson’s disease							
circSLC8A1			Oxidative stress, neuron damage	Brain		Solute Carrier Family 8 Member A1	[70]
circHBB			Response to oxidative stress, hemostasis	Blood	Yes	Hemoglobin subunit beta	[71]
circSIN3A			Response to oxidative stress, hemostasis	Blood	Yes	SIN3 transcription regulator family member A	[71]
circFBXW7			Response to oxidative stress	Blood	Yes	F-Box and FW repeat domain containing 7	[71]
circITGAL			Hemostasis	Blood	Yes	Integrin subunit alpha L	[71]
circ_0004381, circ_0017204				Blood	Yes, early diagnosis		[72]
circ_0085869, circ_0004381, circ_0017204, circ_0090668				Blood	Yes, early-stage from late stage		[72]
circSLAIN1	miR-526b miR-659 miR-1197 miR-516b		Microtubule growth/candidate gene for Intellectual disability	Blood	Yes	Slain motif-containing protein 1	[73]
circDOP1B	miR-659 miR-516b			Blood	Yes	DOP1 leucine zipper-like protein B	[73]
circRESP1	miR-1197 miR-526b miR-578		Neurodegeneration, iron accumulation	Blood	Yes	RALBP1-associated eps domain containing 1	[73]
circMAPK9	miR-526b miR-659 miR-578		Neuronal migration, axonal sprouting, and guidance, neuronal survival	Blood	Yes	Mitogen-activated protein kinase 9	[73]
circPSEN1	miR-1197 miR-526b miR-516b miR-578		Mutations of PSEN1 are associated to early-onset parkinsonism	Blood	Yes	Presenilin 1	[73]
circHOMER1	miR-659 miR-578		Synaptic plasticity	Blood	Yes	Homer scaffold protein 1	[73]

Abbreviations. circFOXO3, circRNA Forkhead box 3; circEP300, circRNA E1A binding protein p300; ciRS-7, circRNA Cerebellar degeneration-related protein 1 antisense RNA; circHDAC9, circRNA Histone deacety-lase-9; circHomer1, circRNA Homer scaffold protein 1; circRTN4, circRNA Reticulon-4; circCORO1C, circRNA Coronin 1; circDOCK1, circRNA Dedicator of cytokinesis 1; circKIF1B, circRNA Kinesin family member 1B; circDLG1, circRNA Discs large MAGUK scaffold protein 1; circAβ-a, circRNA amiloid β-a; circLPAR1, circRNA Lysophosphatidic acid receptor 1; circAXL, circRNA AXL receptor ty-rosine kinase; circGPHN, circRNA Gephyrin; circPCCA, circRNA Propionyl-CoA carboxylase subu-nit alpha; circKCNN2, circRNA Potassium calcium-activated channel subfamily N member 2; circSLC8A1, circRNA Solute Carrier Family 8 Member A1; circHBB, circRNA Hemoglobin subunit beta; circSIN3A, circRNA SIN3 transcription regulator family member A; circFBXW7, circRNA F-Box and FW repeat domain containing 7; circITGAL, circRNA Integrin subunit alpha L; circSLAIN1, circRNA Slain motif-containing protein 1; circDOP1B, circRNA DOP1 leucine zipper-like protein B; circRESP1, circRNA RALBP1-associated eps domain containing 1; circMAPK9, circRNA Mitogen-activated protein kinase 9; circPSEN1, circRNA Presenilin 1.

**Table 2:** circRNAs in aging, Alzheimer’s disease, Frontotemporal Dementia, and Parkinson’s disease experimental models.

CircRNA Name	miRNA Sponge or Mechanism of Action	Target	Possible Pathogenic Role	Experimental Model	Host Gene	References
Aging						
circFOXO3, circEP300		P21, CDK2 proteins/TREM2	Neuronal death, mitochondrial disfunction/neuroprotection	Senescent cell types/Neuronal cells/astrocytes	E1A binding protein p300, Forkhead box 3	[57,74]
circPVT1	miR199a-5p	Let-7/Sirt1		Fibroblasts/tendon stem/progenitor cells	Plasmacytoma variant translocation 1	[75,76]
circCCNB1	miR-449a	Cyclin E2		2BS fibroblasts	Cyclin B1	[77]
circDNMT1	P53, AUF1		Autophagy, inhibits senescence	Breast cancer cell line	DNA-methyltransferase1	[78]
Alzheimer’s disease						
ciRS-7	mir7	UBEA2	promote APP and BACE1 degradation	SH-SY cells	Cerebellar degeneration-related protein 1 antisense RNA	[79]
circHDAC9	mir-142-5p		Neuroprotection/alleviate Aβ-42-induced neuronal damage	Human neuronal cells	Histone deacetylase-9	[80]
circ_0000950	mir-103	PTGS2	Neuroinflammation Neuronal apoptosis, reduced neurite outgrowth	PC12 cells, rat cerebral cortex neurons		[81]
circ_017963			Autophagosome and vesicular transport	SAMP8 mice Brain		[82]
circNF1-419	Dinamin-1 and AP2B1 binding		Mediate autophagy, reduce expression of AD markers and dementia	SAMP8 mice Brain	Neurofibromin 1	[83]
mmu_circ_013636, mmu_circ_012180				SAMP8 mice Brain		[84]
circTulp4			Neurite growth, differentiation and cell viability	APP/PS1 mice	TUB like protein 4	[85]
Parkinson’s disease						
circSLC8A1	miR-128 miR-132	Sirt-1	Neuroinflammation	SH-SY, A53T mouse model, Midbrain dopamine neurons	Solute Carrier Family 8 Member A1	[86]
mmu_circ_000329, mmu_circ_000132, mmu_circ_000597, mmu_circ_000538, mmu_circ_0003292	miR-132	N4A2 PD associated genes		MPTP-induced PD model, mice, brain		[87]
circDLGAP4	miR-134-5p	CREB pathway	Neuroprotection	MPTP-induced PD model, mice, brain SH-SY5Y cells	Discs large Homolog associated protein 4	[88]
circSAMD4A	miR-29c-3p	AMPK/mTOR cascade	Apoptosis and autophagy	MPTP-induced PD model, mice, Brain SH-SY5Y cells	Sterile alpha motif domain containing 4A	[89]
circSNCA	miR-7	SNCA gene	Apoptosis and autophagy	SH-SY5Y cells	Alpha-synuclein	[90]

Abbreviations. circFOXO3, circRNA Forkhead box 3; circEP300, circRNA E1A binding protein p300; circPVT1, *circRNA Plasmacy-toma variant translocation* 1; circCCNB1, circRNA Cyclin B1; circDNMT1, circRNA DNA-methyltransferase 1; ciRS-7, circRNA Cerebellar degeneration-related protein 1 antisense RNA; circHDAC9, circRNA Histone deacetylase-9; circNF1-419, circRNA Neurofibromin-1; circTulp4, circRNA TUB like protein 4; circSLC8A1, circRNA Solute Carrier Family 8 Member A1; circDLGAP4, circRNA Discs large Homolog associated protein 4; circSAMD4A, circRNA Sterile alpha motif domain containing 4A; circSNCA, circRNA Alpha-synuclein.

## Data Availability

Not applicable.
